# Lessons from natural flight for aviation: then, now and tomorrow

**DOI:** 10.1242/jeb.245409

**Published:** 2023-04-17

**Authors:** Christina Harvey, Guido de Croon, Graham K. Taylor, Richard J. Bomphrey

**Affiliations:** ^1^ Mechanical and Aerospace Engineering, University of California, Davis, Davis, CA 95616, USA; ^2^ Micro Air Vehicle Laboratory, Control and Simulation, Faculty of Aerospace Engineering, Delft University of Technology, 2629 HS Delft, The Netherlands; ^3^ Department of Biology, University of Oxford, Oxford OX1 3SZ, UK; ^4^ Structure and Motion Laboratory, Department of Comparative Biomedical Sciences, Royal Veterinary College, Hatfield AL9 7TA, UK

**Keywords:** Aerodynamics, Biomechanics, Bird, Control, Bio-inspired, Insect

## Abstract

Powered flight was once a capability limited only to animals, but by identifying useful attributes of animal flight and building on these with technological advances, engineers have pushed the frontiers of flight beyond our predecessors’ wildest imaginations. Yet, there remain many key characteristics of biological flight that elude current aircraft design, motivating a careful re-analysis of what we have learned from animals already, and how this has been revealed experimentally, as well as a specific focus on identifying what remains unknown. Here, we review the literature to identify key contributions that began in biology and have since been translated into aeronautical devices or capabilities. We identify central areas for future research and highlight the importance of maintaining an open line of two-way communication between biologists and engineers. Such interdisciplinary, bio-informed analyses continue to push forward the frontiers of aeronautics and experimental biology alike.

## Teaser

**Summary:** Biological insights have played a central role in shaping the modern aeronautical field, yet there remain several areas for which a bio-informed approach may offer novel solutions including miniaturization, efficiency and autonomy.

## Introduction

Aviation has been founded upon biological insight since its inception. Leonardo da Vinci's 15th and 16th century notebooks contain sketches of birds and bats, alongside some of the first designs of flying machines (Anderson, 1997). At the end of the 18th century, the notebooks belonging to Sir George Cayley, inventor of the fixed-wing glider, also contain musings on how biological organisms power their locomotion, from caterpillars to herons to sea lions (Cayley, 1793). By the end of the 19th century and turn of the 20th, two sets of siblings would again become interested in bird flight – this time leading them to create flying machines capable of lifting a person. Otto and Gustav Lilienthal were deeply inspired by birds, referencing a fable about a stork teaching a small warbler how to soar (Lilienthal, 1911). After many failed attempts to replicate the stork's flight, Otto ultimately created the first successful hang glider (Anderson, 2018; Lilienthal, 1911) and, later, pushed the design closer to bird flight by incorporating flapping wings. Unfortunately, he was fatally unsuccessful in controlling one of his new gliders. Wilbur and Orville Wright are the best known of all the early aviation pioneers: of the many contributions that enabled them to achieve the first powered flight, a key invention was their lateral control system. Wilbur implied that this was inspired by his observations of buzzards twisting their wing tips, although the brothers disagreed on this account (Anderson, 2002). Yet the impact of bird flight on aviation runs deeper than just these earliest aircraft designs, shaping even the development of subsequent engineering methods. Indeed, the first written appearance of the classic equation determining the lift coefficient was in Lilienthal's seminal book ‘Birdflight as the Basis of Aviation: A Contribution Towards a System of Aviation’ (Anderson, 2018; Lilienthal, 1911).

There is a common thread running through the history of early aviation: aircraft designers observed birds, interpreted what they expected or believed to be how birds flew, and used these insights as inspiration for their own designs ­– and even for the underlying aerodynamic theory. Yet, an equally clear thread running through the following century of aviation design is that most subsequent progress was facilitated by engineering theory and experimentation, rather than by observations of a biological nature. In particular, it was the notion of separating the three key functions of lift generation, propulsion and control that would set the direction of fixed-wing aircraft design for the next century (Anderson, 1997). So it is that most of today's aircraft are supported by rigid lifting wings, are driven by a jet engine or propeller, and are stabilized and controlled by discrete lifting surfaces. This approach contrasts with birds, bats and insects, which flap their wings with a reciprocating motion and morph to effect propulsion and control while simultaneously providing weight support. As a result of these developments inspired by human ingenuity, rather than by nature, there are now fighter aircraft that can take off vertically before transitioning to fly faster than the speed of sound, airliners that can transport hundreds of people halfway around the globe on a non-stop flight from New York to Singapore, and uncrewed multi-rotor aircraft that can perform detailed inspections inside buildings housing nuclear reactors.

Whilst aviation technology made these great leaps forward – driven all too often by the engine of war – the analytical, numerical and empirical tools that engineers had developed for aerodynamic modelling, flow visualisation, control theory and flight mechanics provided a formal foundation for new biological analyses of animal flight. Many of those analyses were published in the Journal of Experimental Biology (JEB), which, over the last century, has led a (sometimes!) more peaceable revolution in modern aerodynamics. In so doing, things have come full circle, because the challenges of modelling complex flight dynamics and unsteady flows are particularly complicated at the intermediate Reynolds numbers associated with flapping flight, such that many cutting-edge advances in computational fluid dynamics (CFD), flow measurement (e.g. particle image velocimetry, PIV) and flight dynamics modelling have been driven by analyses of biological systems. Fluid dynamic phenomena that were first characterized on the wings of animals in the pages of JEB have long since become the domain of specialist fluid dynamicists. For instance, an early paper by two of the present authors used smoke streams generated by burning baby oil on an electrically heated nichrome wire to visualize the vortices over dragonfly wings (Thomas et al., 2004). Although published in JEB, three-quarters of this paper's subsequent citations have been in the physical, mathematical or engineering sciences (Web of Science).

## A symbiotic relationship and framework for new research

This two-way information flow between engineering and experimental biology continues to flourish, with several key challenges in aerospace still standing to benefit from biological insight. Three such domains include: (1) miniaturization, together with the associated challenges of flight through cluttered and potentially gusty environments; (2) enhanced efficiency across different scales and modes of operation; and (3) autonomy, including elements of guidance, navigation and control. Because future engineered systems will not necessarily resemble biological systems closely, and will almost certainly be delivered with the aid of machine learning and optimization, it follows that what will be needed to apply biological insights effectively is what we call here a bio-informed approach. This terminology implies that it is the underlying biological principle that informs the engineering design. It thereby differs from the broader approach of bio-inspired design, where the biological principle underlying the inspiration is often left somewhat vague (e.g. see the discussion of winglets below). It also differs from the narrower approach of biomimetic design, which implies a copy of some biological model, as opposed to abstraction or improvement on an underlying biological principle. Finally, it is distinct from the principle of bio-hybrid design that has recently become popular in the robotics literature (Chang et al., 2020), which incorporates biological elements within engineered systems as a means of enabling the acquisition of structural or material properties that would otherwise be missing from the engineering toolbox.

The bio-informed approach encompasses two key elements: (1) the development of new engineered systems through the embedding of a fundamental biological principle within the engineering design process; and (2) the development of new engineering methods through their application to research aimed at identifying fundamental biological principles. To illustrate what we mean by this, we draw briefly upon two illustrative examples from our own recent work. Our first example relates to the experimental finding that the swooping trajectories of perching hawks are optimized to minimize the distance from the perch at which the wing stalls (KleinHeerenbrink et al., 2022). This biological principle describes how birds learn to fly, but may also prove useful in designing new objective functions for reinforcement learning of perching in autonomous air vehicles with little direct resemblance to birds. Our second example relates to the finding that quadrotors and flapping fliers can use optic flow cues to stabilize their flight without the aid of accelerometers to sense gravity, thereby overcoming the unobservability of body orientation without an accelerometer or a horizon (de Croon et al., 2022). This new principle of engineering design was informed by our understanding of how insects such as bees control their flight, and in turn deepens our understanding of insect flight. Both such elements will continue to shape the aircraft designs of the future, just as they have done to date, drawing deeply upon research published in JEB and other interdisciplinary journals.

## Aims and scope

Despite, but also because of, its position as the leading journal in comparative animal physiology, JEB has long been a microcosm of interdisciplinary science. This is nowhere more true than in JEB's place as the ‘house’ journal for comparative biomechanics. As interdisciplinary approaches have gained traction, animal physiologists now have the tools at their disposal to perform a detailed examination of organismal flight at all levels: from materials, structures and actuators through to sensing, state-estimation, information processing and multi-agent communication. These approaches have been applied to birds, bats and insects, and even to some extinct forms such as pterosaurs. However, to keep the scope of this Review manageable, we focus specifically on the flight mechanics of extant animals, conceptually embedded within their evolutionary context.

Flight mechanics is chosen as a specific subsection of the interface between biology and engineering where the bio-informed design approach offers promise. Accordingly, we do not discuss the physiology of muscles, either natural or artificial, nor biomaterials, or any of the biochemical aspects of flight that might in the future prove relevant to green aviation. This includes discussions related to power density and energy supply, which are important characteristics for small uncrewed air vehicle (UAV) design. Likewise, we do not address the details of sensory transduction, choosing instead to focus on how animals exploit the kinds of sensory information that they obtain compared with air vehicles. For works that capture these components in greater detail, we refer the reader to Altshuler and Srinivasan (2018), Altshuler et al. (2015), Taylor (2005), Taylor and Krapp (2007) and Tobalske (2007, 2016). Rather, we aim to show how a bio-informed approach that fuses biological and engineering knowledge of flight mechanics can yield both a new wave of aircraft engineering design, and a deeper understanding of biological systems. To accomplish this goal, we survey the literature to identify examples of situations where biological insights have been, or could be, instrumental to the advancement of aeronautical design, and where engineering methods have advanced, or been advanced by, our understanding of animal flight.

## Flapping wings

**Fig. 1.**
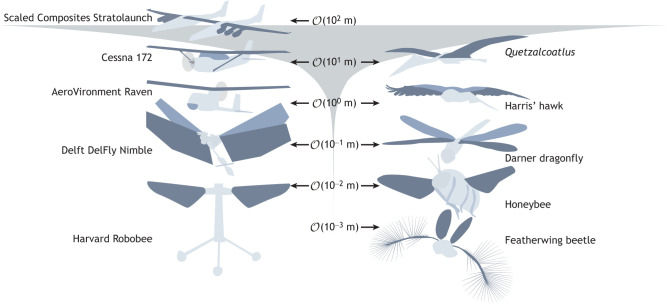
**Flying animals and aircraft visualized at an approximately constant wingspan across several orders of magnitude.** There are no flying animals, extinct or extant, that are on the scale of the aircraft with the largest wingspan. Conversely, there are no flying aircraft that are on the scale of the flying animals with the smallest wingspans, such as the thrips, mymarid wasps and ptilid beetles, whose flight on bristled wings been described in JEB and elsewhere (Ellington, 1980; Farisenkov et al., 2022; Jones et al., 2016). Aircraft (left side, top to bottom) Scaled Composites Stratolaunch, Cessna 172, AeroVironment Raven, Delft DelFly Nimble, Harvard Robobee. Animals (right side, top to bottom): *Quetzalcoatlus*, Harris' hawk, darner dragonfly, honeybee, featherwing beetle. 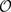, on the order of.

Flying animals and aircraft operate across a broad range of scales (Fig. 1), but there are several key gaps in aircraft design that may, in the future, be addressed by incorporating biological insight. In particular, the use of a pair of flapping wings as a propulsor provides unique advantages in terms of efficiency and manoeuvrability that have attracted the attention of engineers from the earliest days of flight – albeit with limited success until the advent of miniaturization at around the turn of the century. A particular challenge is the effect of scaling on flapping wing designs (i.e. ornithopters, from the Greek: ornis, meaning bird; pteron, meaning wing). At very large wingspans, flapping becomes impractical owing to changes in the flow physics at higher Reynolds numbers and the higher inertial and aerodynamic loading on the flapping mechanism. Flapping wing propulsion becomes viable at smaller wingspans, and it has been suggested that flapping wings will have reduced power consumption relative to a comparable fixed-wing aircraft, especially if using aerodynamic effects that arise owing to wing interactions or wake capture, where the reciprocating wings pass through their own wake (Birch and Dickinson, 2003; Bomphrey et al., 2017; Chin and Lentink, 2016; Nabawy and Crowther, 2014; Pesavento and Wang, 2009; Weis-Fogh, 1973). This effect of scaling means that aircraft can reach much larger scales than flying animals, whilst flying animals reach much smaller scales than aircraft (Fig. 1).

Ornithopters offer one of the clearest examples of how biology has directly inspired aircraft design – albeit in ways that have had little impact on aviation, but considerable impact on those aviators who attempted to support their own body weight using biomimetic or biohybrid flapping wing designs (Anderson, 1997). Roger Bacon wrote about an idea for an ornithopter, and Leonardo Da Vinci sketched multiple ornithopter designs, the majority of which flapped their wings using human power (Goodheart, 2011). Fortunately for the history of Western philosophy and Renaissance art, these aircraft are not believed to have been built, as they would not have been able to fly (Anderson, 2002). In fact, the first human-powered ornithopter to fly straight and level, the Snowbird, was not flown until 2006 (Goodheart, 2011; Robertson, 2010). By that time, the name of this 32-m wingspan aircraft was perhaps the only element of the design that can confidently be said to have derived direct inspiration from birds. For his part, Otto Lilienthal had attempted to incorporate a single-cylinder engine into an ornithopter, but the design proved an unhelpful distraction from the propellor-driven designs that would enable the Wright brothers to make the first successful powered flights, thereby departing almost completely from a bird-like design (Anderson, 2002).

From that point on, engineering studies on flapping flight quickly diverged from studies of biology. Knoller and Betz described how flapping a wing modifies its effective angle of attack, which results in additional lift and thrust components that can both support body weight and balance aerodynamic drag (Betz, 1912; Goodheart, 2011; Jones et al., 1998; Knoller, 1909). Further foundational theoretical developments were contributed in the 1930s by Theodorsen and Garrick (Garrick, 1936; Theodorsen, 1934), but these later texts do not refer explicitly to any biological form of flight, and by this point mathematical proof had long since taken over from biological observation. Nevertheless, in contrast to the unfortunate history of biomimetic and biohybrid ornithopters, these theoretical developments in fluid dynamics offer one of the best examples of the bio-informed approach that we describe. Specifically, a desire to explain the mechanism of thrust production by flapping wings (von Karman and Burgers, 1935) – an observation drawn originally from biology – led to the development of some exquisite aerodynamic theory that still forms the foundation of analytical modelling of aeroelasticity.

At around the same time, biologists were mainly using new experimental techniques to decipher how birds, bats and insects flew. One of the first JEB papers on the mechanics of bird flight was Brown's 1948 paper describing a new high-speed photographic method that could accurately capture the movements of a pigeon's wings while it flapped. This work represents an early step towards quantifying the wing kinematics used by flapping birds, which until then had been too fast to capture. It also details the pigeon's limitations in slow flight, offering insight into the constraints on avian-inspired flapping aircraft. High-speed imaging of animal flight continues to be important to the present day, yielding an ever-richer picture of how animals achieve flight under challenging conditions – for instance, the complex ways in which a bat's wing deforms in flight to enhance flight efficiency (Cheney et al., 2022). Of course, wing kinematics are only one part of the picture, and one of the earliest papers to address the associated aerodynamics was the work in JEB by Osborne (1951), who derived a quasi-steady aerodynamic method to estimate the forces produced by flapping wings based on insect flight. However, owing to the gap between the fields of engineering and biology, this work was not immediately noticed by engineers working on similar topics, and years passed by with a substantial gap between the advancements in the biological and engineering fields on flapping flight.

In the 1960s and 1970s, communication between the two fields was reignited. Pennycuick, who had been a pilot in the Royal Air Force and would later become a Professor in Zoology, made many notable contributions published in JEB, including adapting helicopter theory to model bird flight (Hedenström and Spedding, 2020; Pennycuick, 1968a,b). In 1973, Weis-Fogh proposed the unsteady, high-lift, clap-and-fling mechanism of insect flight in a JEB publication (Weis-Fogh, 1973), which was an important step in solving the so-called ‘bumblebee paradox’ (Bomphrey et al., 2009). This mechanism was of immediate interest to classically trained fluid dynamicists (Lighthill, 1973; Maxworthy, 1979) and was explored experimentally to confirm the hypothesis (Bennett, 1977). Rayner, a mathematician and Professor of Biology, later developed a new vortex theory of animal flapping flight that was explored in published works in both JEB and the Journal of Fluid Mechanics, showing the interdisciplinary nature of the research (Rayner, 1979a,b,c). Many other important contributions on flapping aerodynamics that followed – not least the great body of work on insect aerodynamics by Ellington, who served as Editor of JEB from to 1990 to 1994 (Wootton et al., 2000) and published extensively in the journal (Dudley and Ellington, 1990a,b; Wakeling and Ellington, 1997a,b,c; Willmott and Ellington, 1997a,b). For reviews of this vast literature on flapping flight wing, see Chin and Lentink (2016), Gerdes et al. (2012), Platzer et al. (2008), Sane (2003) and Shyy et al. (2010).

These efforts to reunite the fields were fruitful and have since led to much interdisciplinary cross-pollination, especially regarding the nonlinear aerodynamics of flapping flight. For instance, an extensive body of fundamental fluid dynamics research on leading-edge vortex formation was spawned by work aimed at revealing the aerodynamic mechanism by which insects are able to produce such high lift forces in flapping flight (Ellington et al., 1996). This has been complemented by another very large body of related fluid dynamics research on the efficiency of flapping propulsion. This includes analyses of span efficiency informed by measurements of the downwash in the wake behind insects (Bomphrey et al., 2012; Henningsson and Bomphrey, 2012, 2013; Henningsson et al., 2015) and birds (Henningsson et al., 2014; Usherwood et al., 2020), and the observation that the dimensionless Strouhal number (stroke frequency times stroke amplitude over airspeed) remains approximately constant across a wide range of swimming (Triantafyllou et al., 1991, 1993) and flying (Taylor et al., 2003) animals during cruising locomotion. Both lines of research have taken decades to fully unpick, and have long since become the primary preserve of fluid dynamicists rather than biologists (Taylor, 2018), providing another example of the impact of the bio-informed approach on engineering.

This research on flapping-wing aerodynamics has been facilitated by extraordinary methodological progress in numerical approaches to flow modelling, once again driven by fundamental biological research objectives. In particular, the paper in JEB by Liu et al. (1998) used CFD to model the same leading edge vortex structures that the empirical work by Ellington and colleagues had first observed in hawkmoths and mechanical flappers (Ellington et al., 1996), later validated quantitatively in live insects by direct measurements using particle image velocimetry (Bomphrey et al., 2005). Flapping flight presents a particularly challenging scenario for CFD, owing both to the complexity of the fluid dynamics at intermediate Reynolds numbers including vortex shedding events (Nakata et al., 2015), and the difficulty of accounting for wing deformation. For a significant period, JEB therefore became an outlet in which advances in CFD techniques were not only used but made – all driven by the need to model flapping-wing aerodynamics, which at that time could only be captured by custom-written code. Since then, it has become possible to use off-the-shelf Navier–Stokes solvers to model even the effects of insect wing deformation (Young et al., 2009), and JEB has long since implemented a policy of only admitting computational papers that involve a significant element of experimental biology (Biewener et al., 2012) – another example of the inevitable, and appropriate direction of travel that occurs from fundamental biology to engineering implementation in the bio-informed design paradigm.

Incorporating our fundamental understanding of flapping animal flight into modern ornithopters has been no small feat, and it is no coincidence that one of the most highly cited papers in JEB is a Review on the topic of insect aerodynamics (Sane, 2003). Modern robotics teams have built upon this knowledge to develop advanced flapping wing UAVs. From Delft University's DelFly to DARPA's Hummingbird, many elements of animal flight have been the inspiration for modern UAV designs that would not have arisen otherwise (de Croon et al., 2016; Keennon et al., 2012). Nevertheless, flapping-wing drones are not yet in wide circulation because of the enduring challenges of miniaturization, power economy and flight control. We therefore address ourselves to these general themes, while recognizing that other aspects are also being tackled, such as collision damage mitigation, where concepts are being incorporated that are based on lightweight, deformable insect wing architectures (Mintchev et al., 2017; Mountcastle and Combes, 2014; Tanaka et al., 2022).

## Morphing wings

While flapping wings provide a method of propulsion, biological flight offers further insight into aircraft design if we narrow our focus to gliding flight. Birds use changes in the shape of their wings, tail and body, known as morphing, to adjust their configuration for different tasks, to cope with gusty atmospheric conditions and to perform manoeuvres (Carruthers et al., 2007; Cheney et al., 2021; Gillies et al., 2011; Harvey et al., 2019; Henningsson and Hedenström, 2011; Parrott, 1970; Pennycuick, 1968a; Rosén and Hedenstrom, 2001; Tucker and Parrott, 1970). These shape changes can occur owing to actuation of their muscles or passive deformation of flexible components (Herbert et al., 2000; Smith et al., 2000; Wootton et al., 2000; Young et al., 2009). Insects do not have muscles outside their thorax that can provide active control over the shape of their wings as do birds and bats, but morphing still occurs through torques applied to the sclerites and by passive, prescribed flexibility of their wings (Fabian et al., 2022; Taylor et al., 2012; Wootton, 1992). Such deformations can produce upstroke–downstroke asymmetry and increase the effective stroke angle of the wing tips under inertial loads at stroke reversal, despite limited strain from the flight motor. This flexural property has been useful for minimizing weight from the actuators in Robobee and DelFly flapping aerial robots (de Croon et al., 2016; Wood, 2008). Similar consideration has been given to the influence of bat wing material properties on aerodynamics (Cheney et al., 2022; Hedenström and Johansson, 2015; Henningsson et al., 2018) and inertial power consumption (Fan et al., 2022), and to how those variables will modify the design specification of morphing, bat-like aerial robots (Colorado et al., 2012). However, most studies on active morphing in natural wings tend to be motivated by bird flight, which will therefore be the focus of this section.

Defining what counts as a morphing aircraft is difficult (Barbarino et al., 2011). In principle, we could include any shape change, but this would mean that all current aircraft morph their wings when they lower their flaps. To avoid this wide a grouping, morphing aircraft are often defined as those that perform large-scale morphing (full wing, tail, leg or body changes) or use a non-traditional form of camber morphing. Within this scope, there are many full-scale morphing designs in aviation history, from the warping wings of the Wright Flyer to the variable sweep wings of the Grumman F-14 Tomcat. Although the Wright Flyer's morphing was likely inspired by birds, the F-14 certainly was not. As flight speeds increase towards the speed of sound, increasing the wing sweep is helpful to reduce wave drag, a component of drag caused by the formation of shocks. Birds, of course, do not fly at these speeds, so these considerations are not relevant to the type of flight exhibited by biological flyers. This example highlights how important scaling is in aerodynamics. To make useful aerodynamic comparisons across different conditions, aerodynamicists look to keep consistent similarity parameters, which include the Mach number (ratio of the speed of the flyer to the speed of sound) and the Reynolds number (ratio of inertial forces to viscous forces).

With these scaling effects in mind, there has been a recent shift towards the study of avian morphing with the goal of advancing the design of small-scale UAVs, which operate in a very similar flight regime to birds (Harvey and Inman, 2021). Teams of roboticists, engineers and biologists have designed multiple UAVs that have exhibited unique flight characteristics (Abdulrahim and Lind, 2004; Ajanic et al., 2020, 2022; Chang et al., 2020; Grant et al., 2010; Paranjape et al., 2011). For example, sweep morphing has been characterized in multiple species of birds (Harvey et al., 2021; Henningsson and Hedenström, 2011; Lentink et al., 2007; Pennycuick, 1968a; Tucker and Parrott, 1970) and has been shown to confer useful variation in aerodynamic performance and flight control across different speeds in small-scale UAVs (Ajanic et al., 2020, 2022; Chang et al., 2020; Di Luca et al., 2017).

**Fig. 2.**
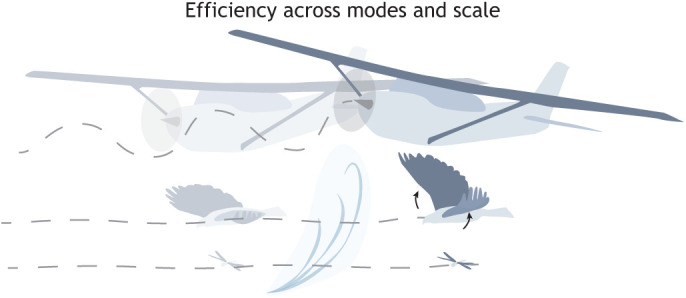
**Animals can adapt effectively to a variety of environmental conditions.** Birds and insects adapt their wing, tail, legs and body morphology to gusts, whereas the vast majority of aircraft remain essentially rigid structures. There is a concerted effort to move towards morphing wings at the frontiers of aerospace research and testing in the expectation that this will widen the flight performance envelope or operating conditions.

Sweep-morphing will also have a substantial impact on a flyer's dynamic characteristics. This includes stability, which is the tendency for a flyer to return to its equilibrium position after a disturbance, such as a gust (Thomas and Taylor, 2001). The majority of birds can shift between a stable and unstable flight configuration by morphing just their elbow and wrist joint (Harvey et al., 2022a). However, control of these distinct dynamic states is difficult to implement on UAVs, so more research into adaptive control strategies is necessary before this can become a reality (Ajanic et al., 2020). This is a worthwhile pursuit, because morphing allows birds to adjust their response to perturbations as well as to effectively manoeuvre, so UAVs that can be effectively controlled across these states may be able to achieve bird-like manoeuvrability and gust response (Harvey and Inman, 2022). Similar capabilities can be gained from wings that vary passively in thickness and camber with flight speed (Cheney et al., 2021), or that articulate passively over large angles at the shoulder prior to an active recovery phase that involves more dramatic shape changes (Fig. 2) (Cheney et al., 2020; Reynolds et al., 2014). For further details on recent advances in morphing aircraft design, we direct readers to previous review papers (Barbarino et al., 2011; Harvey et al., 2022b; Li et al., 2018).

**Fig. 3.**
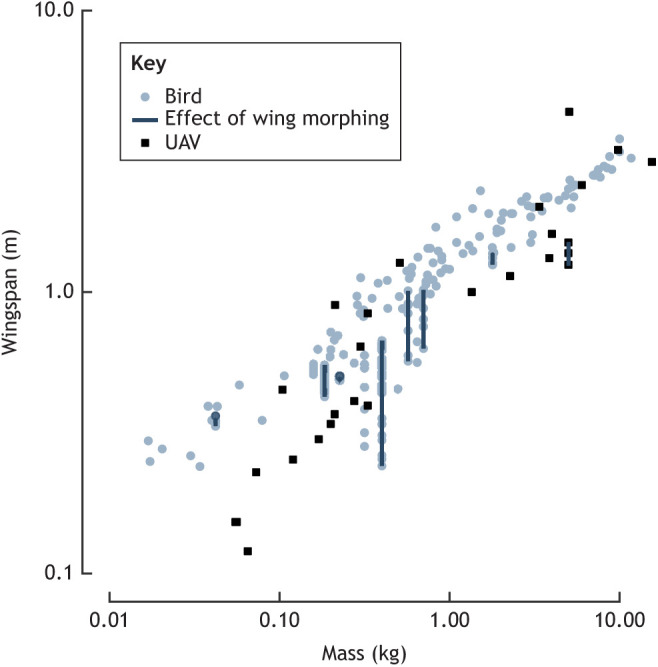
**Small birds fly with higher wingspans than fixed-wing UAVs of comparable mass.** Moreover, the wingspan is reconfigurable over very short timescales during flight, owing to the degrees of freedom inherent in the musculoskeletal anatomy of the shoulder, elbow and wrist joints. Morphing the wing in this way can change the wingspan very substantially. Adapted from Harvey and Inman (2021).

Although there are many remaining unknowns in birds' usage of morphing in flight, there is another related aspect that should not be overlooked in future UAV design. When we compare mass with wingspan across a variety of birds and UAVs, we find that UAVs often fly with smaller wingspans than birds of the same mass (Fig. 3) (Mohamed et al., 2022). The low wingspan used by UAVs is in part a result of designing UAVs with lower aspect ratios for improved efficiency in subcritical Reynolds number regimes (Harvey and Inman, 2021), but by incorporating morphing, birds span a broad range of this design space. By quantifying the aerodynamic characteristics of birds at low Reynolds numbers, such as owls in slow forward flight (Usherwood et al., 2020), it may become possible able to identify novel design approaches that would permit the use of larger wingspans in these low Reynolds number regimes.

## Aerofoils

**Fig. 4.**
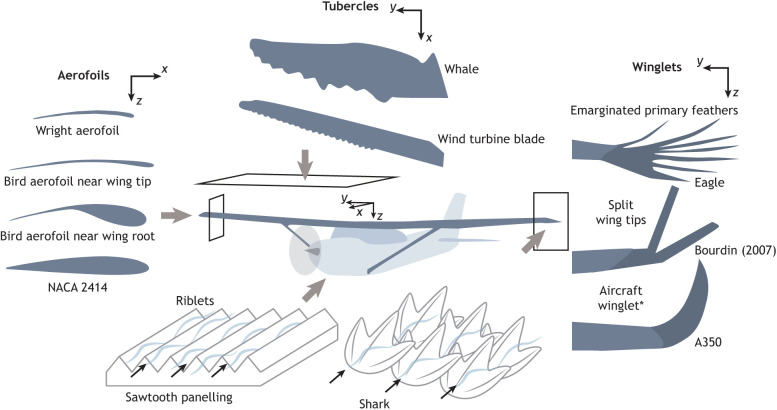
**Multiple biological features have inspired advancements in aircraft or aeronautical design.** Aerofoils began as inspired by the thin bird wing tip. Tubercles have led to advancements in wind turbine blade technology. Winglets, although not directly inspired by birds, can be compared with raptor emarginated primary feathers, and recent studies have considered split wing tips. Riblets have been suggested as a method to control the surface texture of an aircraft, simplifying from the complex nature of scales. *Note that there are many different possible configurations of aircraft winglets.

The cross-sectional shape of aircraft wings and tail surfaces is known as an aerofoil (or aerofoil). In 1884, Horatio F. Phillips was inspired by the shape of bird wings and patented the first cambered aerofoil (Anderson, 2002). Thin cambered profiles were practical to implement in the cambered fabric skins of early aircraft, including those built by the Wright brothers (Fig. 4), and the conventional wisdom of the time was that as birds also had thin aerofoils, these would be superior to thicker aerofoils (Anderson, 2018). Yet, this casual observation and conclusion are both only partially correct. Whilst it is true that the distal sections of bird wings are formed by thin feathers, the proximal section of a bird's wing has bones, tissue and musculature near its leading edge, which leads to a thicker aerofoil over a large portion of a bird's wing (Bachmann, 2010; Carruthers et al., 2010; Cheney et al., 2021; Hieronymus, 2016; Nachtigall and Wieser, 1966). These differences affect the pressure distribution (Usherwood, 2009; Usherwood et al., 2003, 2020). Moreover, engineers soon discovered that the thinner aerofoils were not the most aerodynamically optimal design (Anderson, 2018). Detailed studies characterizing aerofoil properties revealed that a thicker section with a more rounded leading edge helps to delay flow separation, allowing an aircraft to attain higher angles of attack, and thus lift, prior to stall.

This provides a direct example of how bio-inspiration that is not informed by knowledge of the actual biological system may be misleading. Had engineers considered the thickness of bird wings in the first place, it may have been possible to improve aerofoil design sooner. Nowadays, there are multiple ways to optimize aerofoil shape to fit desired characteristics and the need for avian inspiration has largely declined, yet methods of actuation and the materials used to deliver a continuous surface of the required material properties are clearly far removed from evolved biological solutions.

Recently, researchers have returned to studying the aerofoils on bird wings to gain inspiration for small UAVs including micro aerial vehicles (MAVs). With this goal, novel bird-like aerofoils were designed informed by previous experimental studies that quantified bird's characteristics including Withers’ (1981) paper published in JEB, among others (Ananda and Selig, 2018; Klaassen van Oorschot et al., 2016; Liu et al., 2006; Nachtigall and Wieser, 1966). These new aerofoil designs have already been implemented successfully (Moulton and Hunsaker, 2021; Savastano et al., 2022). Building on this momentum, future collaborative work between biologists and engineers may further reveal how advantageous, adaptive, aerodynamic performance can be achieved using bird-like aerofoils.

## Winglets

Winglets are an additional surface attached at some angle to the wing tip of an aircraft and serve to interrupt the formation of the wing tip vortex, which reduces the induced drag of a wing (Fig. 4). They are sometimes discussed as an example of bio-inspiration owing to their similarities with raptor primary feathers that deflect upwards during soaring. However, winglets were nearly exclusively designed based on fundamental fluid dynamical insights. The first patent for a winglet-like device was in 1897 from Lanchester, borne out of aerodynamic theory, and this early insight was popularized by the National Aeronautics and Space Administration (NASA) in the 1970s (Whitcomb, 1976). Now many modern aircraft use winglets for efficiency improvements with a constrained wingspan. Even so, biological insight remains important to novel winglet design. Much of the biological work has focused on the role of the slots between these feathers. For example, Tucker (1993) estimated that the slots would reduce the induced drag on the wing similar to the effect of winglets. A comparable implementation has been split-wing tip designs (Fig. 4), that have recently been explored with renewed interest (Hui et al., 2021; KleinHeerenbrink et al., 2017; Lynch et al., 2018).

In the engineering realm, there has also been a recent push to develop articulated control of winglets, although birds do not have active control over the deformation of their feathers. Instead, a more direct comparison is Airbus' AlbatrossONE concept, which uses passive hinges on the wing tips to reject gust disturbances (Wilson et al., 2019), analogous to the wing-tucking flight behaviour of birds (Reynolds et al., 2014). Intriguingly, this application complements recent experimental biological work that has found that bird wings can act as a passive suspension system minimizing the effects of a gust on the bird's body trajectory (Cheney et al., 2020). In practice, the mechanisms at play are fundamentally different, because the latter system relies on the mass of the wing being elevated under increased loads (as an interaction with the centre of percussion) when decoupling the centres of mass of wing and body/fuselage. It follows that there is likely to be considerable scope in this domain for a more closely bio-inspired approach.

## Tubercles

Birds are not the only animals that have inspired advances in aeronautical capabilities. Swimming animals provide insights into fluid dynamic phenomena that may be implemented in air applications. In 1991, Dennis M. Bushnell, now a Chief Scientist at NASA Langley, wrote a review paper focused on drag reduction techniques in nature (Bushnell and Moore, 1991). This work proposed that the leading-edge bumps (known as tubercles) on humpback whales' pectoral fins should be explored in more detail as they could play a role in drag reduction (Fig. 4). Fish and colleagues took up this challenge and completed a series of experiments investigating the role of tubercles on whale fins (Fish and Battle, 1995; Miklosovic et al., 2004). Their findings indicated that the tubercles' contribution to drag reduction was minor. Instead, the tubercles led to higher angles of attack prior to stall and higher maximum lift coefficients. Of note, the team noted that the humpback whale is the only baleen whale that relies on manoeuvrability while capturing its prey, hinting at a possible role of tubercles in dynamic situations (Hain et al., 1982; Miklosovic et al., 2004). With this biological insight, engineering studies have since confirmed that tubercles act to delay or control dynamic stall (Hrynuk and Bohl, 2020). The mechanism for this improved response is attributed to streamwise vortices that develop off of the crests of each tubercle. These vortices add energy to the flow, allowing the underlying flow to remain attached even at high angles of attack and, thus, reduce the likelihood of both dynamic and static stall (Hansen et al., 2010, 2011; Hrynuk and Bohl, 2020; Watts and Fish, 2001).

Integrating this scientific and engineering fundamental knowledge indicated a reduced need for tubercles in large-scale aircraft design, given that most aircraft avoid operating near stall conditions, yet tubercles have been implemented in wind turbines (Fig. 4) because these devices regularly operate over a wide range of angles of attack (Ibrahim et al., 2015; Kumar and Amano, 2012). Therefore, the study of tubercles offers a fantastic example of how open lines of communication between engineers and biologists can lead to substantial advancements in both fields. First, engineering insight suggested that more biological knowledge was needed. Then, the resulting biological studies identified a use case for the mechanism in animals. Finally, engineers developed a mechanistic understanding of the effect of a wavy leading edge for wing design and used this understanding to implement tubercles on a system that would most benefit from its installation.

## Riblets

Thus far, we have discussed biological inspiration that has affected the structure, kinematics or geometry of aircraft. However, biological inspiration has contributed to developments in materials and micro-scale structures too. One common example is the role of riblets, inspired by fish and shark skin. Riblets are fine surface striations, often implemented as sawtooth grooves oriented parallel to the incoming flow (Fig. 4). Riblets provide an interesting example of convergent evolution in the design process. The role of fish skin in aerodynamic flow control was proposed in the biological community as early as 1969 (Burdak, 1969). Concurrently, engineers began investigating the role of fine structures inspired by geometry studies on heat exchanger fins (Bushnell, 1978; Kennedy et al., 1973; Walsh, 1983). These two approaches converged and many mechanistic studies have since found that grooves effectively reduce skin friction drag and may enhance thrust on a flapping aerofoil (e.g. Oeffner and Lauder, 2012). These findings have led to novel materials, coatings and structures ranging from aerospace applications to swimsuits (Wen et al., 2014). For aircraft applications, it is difficult to manufacture a large surface with such fine microstructures, although previous flight test studies found that this would be beneficial for drag reduction (Walsh et al., 1989). More recently, the Japan Aerospace Exploration Agency (JAXA) developed a paint that could recreate these patterns without the additional complexity of manufacturing special panels. They performed wind tunnel testing supported by flight tests on a full-scale aircraft to support the use of such a method for drag reduction in aircraft (Kurita et al., 2018). Although this progress is exciting, there is still much work required to bring such a unique approach to large-scale operations.

## Information gathering and processing

Another area in which there is a wide divide between engineering efforts and biological reality is that of information gathering and processing. In the fields of artificial intelligence and robotics, even the goals of processing are heavily debated, as exemplified by the task of navigation. In what is currently the dominant approach to autonomous navigation, a three-dimensional model of the world is seen as a primary goal of visual processing. Consequently, robots build highly detailed three-dimensional maps of the world, requiring large amounts of computer memory and processing. In contrast, the navigation strategies of animals with small brains, such as honeybees, suggest that a bio-informed approach to engineering design may bring advantages in terms of efficiency and robustness. For example, studies have shown that bees can successfully navigate cluttered obstacle fields under a variety of wind conditions (Burnett et al., 2020, 2022).

**Fig. 5.**
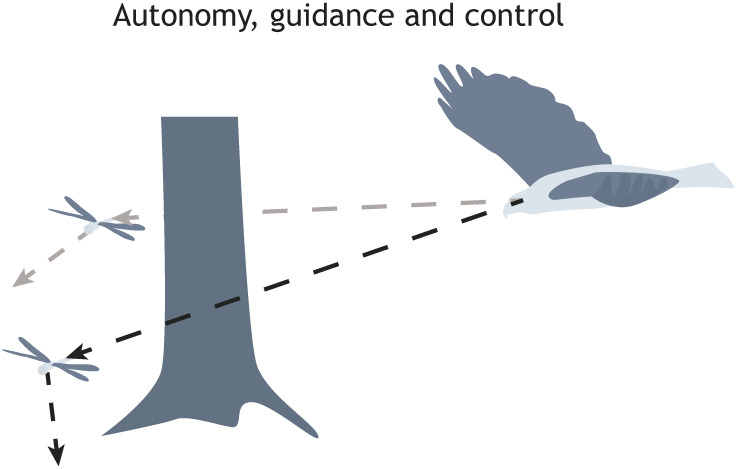
**Guidance, navigation and control are essential for performing everyday tasks, including hunting and evasion.** Here, a hypothetical hunting bird pursues a dragonfly around a tree trunk representing a cluttered environment. Performance demands are greatly increased owing to the added requirements of avoiding collisions with obstacles while simultaneously delivering movement along an adaptive interception trajectory. Stabilization mediated by visual processing of the local visual environment could be complicated by a moving background and strong parallax effects, and targeting is likely to be more challenging if there is only an intermittent line of sight between predator and prey.

There are many examples of exceptional sensitivity in animals. These include the detection of tiny angular displacements of insect antennae in response to body rotations or self-induced airflows (Johnston, 1855; Sane et al., 2007), and the echolocating abilities of bats (Hartridge, 1920). Such examples point to new ways of achieving key capabilities in drones such as nocturnal collision avoidance (Laurijssen et al., 2019; Nakata et al., 2020). These are important for a range of guidance, navigation and control tasks both in animals and in robots that mimic their capabilities (Fig. 5). However, in contrast to aircraft, which typically use a handful of high-quality sensors arranged in an orthogonal fashion, flying animals commonly make use of a vast array of sensors arranged in a distributed and highly non-orthogonal fashion (Land and Fernald, 1992; Sterbing-D'Angelo et al., 2017; Taylor and Krapp, 2007) Hence, whereas aircraft sensors are calibrated to provide precise and accurate estimates of the vehicle's motion state (i.e. its position, orientation, velocity and angular velocity), the sensors of flying animals are set up as uncalibrated feature detectors. This is suggested to relate to a different underlying principle of sensorimotor organization, called the mode-sensing hypothesis, wherein the sensory systems of insects may be tuned as matched filters adapted to detect excitation of their natural modes of motion (Taylor and Krapp, 2007).

Modes of motion describe the ways in which a dynamical system characteristically moves (e.g. the periodic swing of a pendulum). Understanding these dynamics is key to the design of any vehicle control system, whether its sensors are set up as matched filters as the mode-sensing hypothesis proposes. A substantial body of work on insect flight in JEB has therefore been dedicated to elaborating the natural modes of motion of insects by borrowing approaches to modelling flight dynamics from engineering across to biology. The first of these papers (Taylor and Thomas, 2003) used the linearized equations of motion commonly used in aircraft flight dynamics modelling to model the flight dynamics of locusts, with equations parameterized using empirical measurements from live insects flying tethered to a wind tunnel force balance. This was followed soon after by a series of papers that used CFD to parameterize the equations of motion to the same end, confirming the important result that insects are inherently unstable in flight, and therefore require the use of sensorimotor feedback to fly at equilibrium at all (e.g. Sun and Xiong, 2005).

One of the primary sources of sensory feedback in insect flight is visual processing, and a key topic in visual processing that has been studied intricately in both engineering and biology is that of optic flow. Hence, although there are other kinds of sensors, notably airflow-sensitive hairs, that hold considerable future promise for the bio-informed approach, optic flow processing offers the single best example of how the study of information gathering and processing in animals has informed the design of autonomous systems including small drones. Optic flow is the apparent motion of objects in the world caused by the relative motion between these objects and an observer. This motion is apparent by means of angular changes to these objects over the retina or facet eyes. Although von Helmholtz had already mentioned ‘variations of the retinal image due to bodily movements’ in the context of depth perception (von Helmholtz, 1925), the concept of optic flow was first introduced by psychologist Gibson, and was an important element of his ecological approach to cognition, in which perception revolves around affordances for action (Gibson, 1950). Further work has elucidated in detail the neural mechanisms that underpin this capability since pioneering work by Reichardt (1961) and others since (Krapp and Hengstenberg, 1996; Schuster et al., 2002; Strauss et al., 1997; Wolf and Heisenberg, 1986). Indeed, the optic flow field provides various types of behaviourally relevant information on ego-motion and time-to-contact, which is a first-order approximation of the time it will take for the observer to touch or pass an object. The time-to-contact illustrates the important property of optic flow that it only carries information on the three-dimensional structure of the scene relative to the velocity of the observer. Hence, distance and velocity are intertwined in optic flow measurements.

Animals seem to use optic flow cues for controlling many important behaviours. Early studies showed how gannets decide when to streamline while plunging based on time-to-contact (Lee and Reddish, 1981) and how flies determine when to decelerate for landing on a target (Wagner, 1982). Perching pigeons were found to keep the time derivative of the time-to-contact from their feet to the perching object constant (Lee et al., 1993), and also use optical cues to negotiate their way through obstacles by steering through gaps (Lin et al., 2014). Honeybees keep the ventral optic flow magnitude constant for grazing landings (Srinivasan et al., 2000) and the image rate of expansion constant for vertical landings (Baird et al., 2013); they also balance the optic flow sensed in their left and right visual hemispheres in order to centre themselves when flying down tunnels (Srinivasan et al., 1996). The potential use of optic flow control for flying robots was soon recognized by biologists (Franceschini et al., 1992; Srinivasan et al., 2001), leading to the implementation of optic flow control loops on flying robots for landing, flying over undulating terrain, and avoiding obstacles (de Croon et al., 2021; Herissé et al., 2012; Hyslop and Humbert, 2010; Ruffier and Franceschini, 2004; Ruffier et al., 2003; Serres et al., 2008). This line of bio-informed research turned out to be very useful when drone manufacturers started to develop drones for indoor use. Whereas outdoors, vehicles can use the satellite-based global positioning systems to obtain both location and velocity information, such information is not available indoors. Currently, the standard solution for indoor drones is to use optic flow from a downward-looking camera to obtain velocity estimates. However, because optic flow only captures the ratio of velocity and height which must be scaled in some way, the drones carry an additional distance sensor (sonar, laser altimeter or otherwise) to scale the optic flow for obtaining velocity.

Robotics research on optic flow also offers new hypotheses about optic flow control by animals. For instance, Hérissé and colleagues demonstrated early on that vertical landings of a hovering drone could be performed successfully with the help of optic flow divergence (Herissé et al., 2008), which was later found to be the vertical landing strategy employed by honeybees (Baird et al., 2013). Robotic studies have also revealed that controlling constant optic flow landings is more challenging than it seems. In particular, it turns out that successful optic flow landing requires the control gains (the proportional relationship between the sensory input signal and the controlled motor output signal) to scale with the distance to the landing object (de Croon, 2016), but such information is not readily available from optic flow. Multiple approaches have been proposed in which robots and animals can successfully control optic flow landings without the need for additional scaling sensors. van Breugel et al. (2014) found that during a constant optic flow landing, the distance can be determined based on the control action (such as a motor power reference value) in combination with the optic flow divergence and its rate of change.

De Croon proposed that robots and insects can exploit the (in)stability properties of the optic flow control loop for determining distances (de Croon, 2016). When approaching an object, control actions will lead to increasingly large angular and hence optic flow changes. Consequently, a fixed-gain optic flow controller will generate increasingly strong control actions, leading to self-induced oscillations, that can be used to trigger a landing response (de Croon, 2016), to refine optic flow control (Ho et al., 2018) or to improve distance estimation based on visual appearance (de Croon et al., 2021). Finally, a forward model (Webb, 2004), that uses prior knowledge of the likely sensory input that will be received during a change in motion, can be used to scale optic flow measurements over time (Bergantin et al., 2021; Ho et al., 2017). Many of these contributions derive from the use of robots as models. These physical models are particularly interesting because they cannot overlook properties of the real world. However, they can only generate hypotheses about the natural world, which must subsequently be tested by experiments with animals. Such experiments can be extremely challenging, as the hypotheses often pertain to internal brain processes.

This brings us back to the nature of how gathered information is processed (or appropriately transforms sensory inputs to provide signal outputs). Many bio-inspired robotics studies currently model processing at a high level of abstraction. Whereas processing in animals happens through complex chemical and electrical processes, modern computing does not use architectures based on biochemistry and, thus, most robot controllers involve sequential algorithms executed on classical von Neumann processing architectures. For instance, in finite state machines, states represent behaviours and transitions link together these behaviours. This enables fast execution of composite behaviours on traditional processors, such as the casting and surging of fruit flies to find odour sources (Anderson et al., 2019, 2020; Barrows, 1907; Lochmatter and Martinoli, 2009; Saxena et al., 2018; van Breugel and Dickinson, 2014).

In contrast, artificial neural networks (ANNs) more closely mimic the nature of biological processing, i.e. parallel processing by a large number of neurons that individually perform limited functions but collectively can perform highly complex functions (McCulloch and Pitts, 1943; Rosenblatt, 1958; Schmidhuber, 2015). It is widely acknowledged that common ANNs are only very loosely inspired by biological neurons, as they represent much simpler functions than those performed by natural neurons. Although this has not prevented great achievements in the field of artificial intelligence (AI) (Jumper et al., 2021; Perolat et al., 2022; Silver et al., 2017), recently, the drive for edge AI (where data from the physical world are processed locally rather than centrally, and often close to the sensory apparatus) has spurred an increasing effort into the development of a closer approximation of the dynamics of biological neurons. Spiking neural networks (SNNs) have temporal dynamics more similar to natural neurons, including a membrane voltage that causes a spike when exceeding a threshold (Maass, 1997). Owing to the binary nature of spikes, SNNs hold the potential of orders of magnitude lower latency and more energy efficiency than traditional ANNs. This makes SNNs especially promising for small flying robots that have to react quickly to their environment, while being inherently constrained in terms of energy. However, designing and training SNNs is currently still much more challenging than for ANNs (Tavanaei et al., 2019). These challenges are due to the discontinuous nature of the spiking function, the more complex neural dynamics that can lead to saturation or dwindling of neural activity, and the higher-dimensional parameter space defining the neurons. Hence, currently, robotic implementations of neuromorphic processing are still quite limited in complexity (Bing et al., 2018).

## Concluding remarks

As Lilienthal (1911) recognized, biological insight has played, and will continue to play, a foundational role in advancing aviation. Here, we have discussed some of the varied ways in which key contributions to fundamental biological knowledge, many of them published in JEB, have advanced engineering design and engineering methods. Nevertheless, there remains a clear gap between the capabilities of animals and those of modern aircraft. Areas warranting exploration and quantification include the dynamics and control of specialized flight morphologies, including those associated with miniature and morphing-wing flight, together with the very different information processing and sensorimotor architectures that animals use compared with aircraft.

Biological insight into these areas could lead to enhanced performance by enabling navigation through cluttered environments, adaptation to unpredictable events, and system resilience in the face of gusts, turbulence and damage. However, the extent to which bio-informed design may contribute to solving these problems will depend on the extent to which insight can be successfully incorporated within the machine learning and optimization approaches that will be used to design the aircraft systems of the future. First attempts at this have been promising, and it is noteworthy that one of the first successful uses of reinforcement learning on an autonomous vehicle (Reddy et al., 2018) was developed in the context of soaring flight inspired by birds (Reddy et al., 2016). In conclusion, the most effective way forward will be to foster ever deeper relationships between biologists and engineers with genuine two-way knowledge exchange, and by training new members of the research community who no longer recognize these silos and, instead, stand astride the shoulders of both these traditional fields.
